# Informational Analysis for Compressive Sampling in Radar Imaging

**DOI:** 10.3390/s150407136

**Published:** 2015-03-24

**Authors:** Jingxiong Zhang, Ke Yang

**Affiliations:** School of Remote Sensing and Information Engineering, Wuhan University, 129 Luoyu Road, 430079 Wuhan, China; E-Mail: keyang@whu.edu.cn

**Keywords:** compressive sampling, rate distortion, mutual information, complex-valued scenes, radar imaging, under-sampling ratios, Gaussian mixtures

## Abstract

Compressive sampling or compressed sensing (CS) works on the assumption of the sparsity or compressibility of the underlying signal, relies on the trans-informational capability of the measurement matrix employed and the resultant measurements, operates with optimization-based algorithms for signal reconstruction and is thus able to complete data compression, while acquiring data, leading to sub-Nyquist sampling strategies that promote efficiency in data acquisition, while ensuring certain accuracy criteria. Information theory provides a framework complementary to classic CS theory for analyzing information mechanisms and for determining the necessary number of measurements in a CS environment, such as CS-radar, a radar sensor conceptualized or designed with CS principles and techniques. Despite increasing awareness of information-theoretic perspectives on CS-radar, reported research has been rare. This paper seeks to bridge the gap in the interdisciplinary area of CS, radar and information theory by analyzing information flows in CS-radar from sparse scenes to measurements and determining sub-Nyquist sampling rates necessary for scene reconstruction within certain distortion thresholds, given differing scene sparsity and average per-sample signal-to-noise ratios (SNRs). Simulated studies were performed to complement and validate the information-theoretic analysis. The combined strategy proposed in this paper is valuable for information-theoretic orientated CS-radar system analysis and performance evaluation.

## 1. Introduction

Compressed sensing (CS) is a new methodology for information acquisition and processing, as it provides a framework for directly acquiring data already in compressed form (rather than the conventional sampling-compression practice [1]), thus promoting under-sampling or sub-Nyquist sampling strategies that are more efficient than what is required by the Shannon–Nyquist sampling theorem [2,3]. It has been applied to various fields, including optical and radar remote sensing [4–11]. CS works on the assumption of the sparsity of the scene being sensed, relies on the informational transferability of the sensing/measurement matrices in capturing the information content in the underlying signal (or scene in the context of radar) and operates through algorithms that can reconstruct the sparse signal from under-sampled data [7,12–16].

As hinted above, information theory is fundamental to the understanding and analysis of CS and CS-based systems, such as CS-radar [6,7], as well as conventional radar [17–20]. The reason is that information theory provides theoretic explanations of CS mechanisms and describes performance limits of a CS-based system better than otherwise, because “information”, rather than “data”, is the essence of CS. For example, we can examine signal sparsity or compressibility (so, there has been much work on image compression [1,21] before the advent of CS), measurement matrices, signal reconstruction and other elements in a CS context based on informational analysis [22–24]. Furthermore, information-theoretic principles provide a basis complementary to established CS theory for the derivation of necessary and sufficient conditions on sampling rates in CS, which are termed undersampling ratios, because of their sub-Nyquist nature [9,25]. For example, Fano inequality, rate distortion and the channel coding theorem are often applied for undersampling theorem developments [26–28], while statistical analysis of the signal reconstruction process (e.g., error probability bounds in signal reconstruction, especially when used in connection with Fano inequality) is also an important ingredient [29].

The majority of published work on CS sampling and its informational aspects is based on the assumption of the randomness of measurement matrix ensembles [27–32]. However, those involved in radar are often deterministic, because they are prescribed by the specific filters involved (as will be described in the next section). As shown by Alonso *et al.* [5], by construction, the convolution matrix in radar echo signal modeling is a band matrix (the column vectors are the samples of the transmitted waveforms, which are often chirp signals), where the product between columns decreases with increasing distance between the columns (implying decreasing coefficients of correlation). This suggests limited transferability of the published results about CS sampling rates to CS-radar. Although Aeron *et al.* [33] describes how necessary CS sampling conditions may be derived in situations where deterministic measurement matrices are employed, their results are not directly applicable for radar imaging due to the generally non-standardized forms of radar measurement matrices and the complexity of complex-valued distributions involved in radar signal modeling [34–38].

In addition to theoretical approaches to determining CS undersampling ratios, the other way is by simulation-based experiments (*i.e.*, computational experiments). The theoretical and computational methods behave as deductive and inductive methods do, with the latter being more computationally expensive (in exchange for being more versatile in dealing with peculiar signal models, sampling matrices, reconstruction algorithms and performance evaluation criteria, which are often difficult to analyze theoretically). Their complementarity should thus be explored. For example, the former can provide analytical expressions for undersampling-sparsity relationships, which can be further validated and enhanced by the computational results of the latter.

Our work is motivated by the advantages of combining theoretical derivation and computational validation for informational analysis and sub-Nyquist sampling theorem development in CS-radar imaging. This paper seeks to describe, analyze and interpret information flows in CS-radar imaging from the perspective of information theory. Informational analysis focuses on compressibility (of radar scenes or images), trans-information of radar measurements about the underlying scene (*i.e.*, mutual information between radar measurements and the scene) and information-theoretic derivation of necessary undersampling ratios for signal reconstruction. In addition to theoretical derivations, simulation-based experiments will be performed to demonstrate informational characterization of a hypothetical CS-radar system and to validate analytical results derived from use of information theory.

In summary, the proposed information-theoretic methodology is complementary to classic CS theory for CS-radar system performance analysis and sampling design, offers some advantages in terms of the ease of implementation and provision of an informational quality indicator (upper bound) for the reconstructed image and is effective, as confirmed by simulation-based validation. The novelty of the paper is two-fold: (1) proposing a general strategy, complementary to classic CS theory, for information-theoretic analyses and undersampling theorem developments in CS-radar; and (2) promoting the combined use of information-theoretic derivation and computational validation for CS-radar information analysis and sampling necessity determination using a hypothetical, yet representative radar imaging example. While the second aspect of novelty has been discussed previously, the first claim of novelty is due to the fact that, despite existing literature on the themes of information theory and CS, information theory and radar, and CS-radar, work on integrating all three areas (information theory, CS and radar) is rare, and few existing results are information-theoretic orientated and directly applicable to CS-radar. This paper bridges the gap between information theory and CS-radar by analyzing information dynamics in CS-radar and determining sub-Nyquist sampling rates necessary for scene reconstruction.

Major contributions of the paper include:

(1) proposing general formulas for quantifying trans-information of radar measurements about the underlying scene (accounting for the often deterministic nature and non-standardized form of measurement matrices in radar measurements), which can be used both for determining necessary undersampling ratios for image reconstruction and as an informational quality index (upper limit) for the resultant image reconstructed from undersampled data,
(2) clarifying the use of complex-valued Gaussian mixture distributions for modeling strictly or approximately sparse radar scenes and suitable rate distortion functions, and
(3) promoting the complementarity of theoretical analysis and computational methods for CS-radar sampling theorem development and experimental validation, which are crucial for information-theoretic optimized CS-radar system designs and applications.

We do not claim that the work and findings reported in this paper are ground-breaking in theoretical terms, but assert that they are constructive to further developments in the interdisciplinary field of information theory, CS and radar. To reiterate, the research reported in this paper is significant, given the facts that cost-effectiveness will remain an issue for remote sensors, even when we are increasingly resourceful in terms of storage and computing, and information theory is fundamentally important for sustainable developments in CS-radar, where it is not mere data, but information (in terms of Shannon information) that matters.

Below, after a description of some radar fundamentals, compressible radar scenes are modeled via Gaussian mixture distributions, and their rate distortion functions are discussed. This is followed by a description of the method for determining mutual information (upper bounds, to be exact) between compressive measurements and the underlying scene, which measures the amount of information conveyed by such measurements about the scene. Necessary sampling ratios are determined by requiring the amount of trans-information being at least as large as that of rate distortion. Based on the descriptions of the models and methods, a simulation-based experiment is then reported, with results discussed and compared with some relevant ones in the literature. Lastly, some concluding remarks are given.

## 2. Models and Methods

### 2.1. Radar Imaging: Traditional vs. CS Strategies

A synthetic aperture radar (SAR) synthesizes the coherent pulses during its integration time to produce radar images at high spatial resolution. Consider a strip map mode SAR with a single channel. A chirp signal (*TRN*(τ)) is usually adopted as the transmitting signal for a radar system [39]:

(1) $$\begin{array}{cc} \text{TRN}(\tau )=\mathrm{rect}\left(\tau /T_{p}\right)\exp\left\{J\pi K_{r}\tau^{2}\right\}, & \tau \in \left(-T_{p}/2,T_{p}/2\right] \end{array}$$

where τ is the fast time, *T_p_* represents the time duration of the chirp pulse, *K_r_* is the chirp rate, rect(.) stands for the rectangular function and *J*^2^ = −1.

For a strip map SAR, the radar platform moves in the azimuth direction (slow time direction), and the antenna illuminates the scene and receives the echoes reflected from therein. The echo signal can be written as:

(2) $$Y\left(\tau ,\eta \right)=\iint_{x,y}X\left(x,y\right)w_{a}\left(\eta -x/v\right)\exp\left\{-J4\pi f_{0}RG\left(x,y,\eta \right)/c\right\}\cdot \text{TRN}\left(\tau -2RG\left(x,y,\eta \right)/c\right)\text{d}x\text{d}y+N\left(\tau ,\eta \right)$$

where τ is the fast time, η is the slow time, (*x*, *y*) indicates the azimuth and range position of a target, *X*(*x*, *y*) is the backscattering coefficient at (*x*, *y*), *w_a_* is the azimuth weighting function, *f*_0_ is the carrier frequency, *RG*(*x*, *y*, η) is the slant range, *v* is the platform velocity relative to the ground, *c* is the speed of light and *N* is the thermal noise at the receiving terminal [9,39]. The equation above can be simplified as:

(3) Y(τ,η)=A(τ,η,x,y)⊗X(x,y)+N(τ,η)

where *A*(τ, η, *x*, *y*) = *w_a_*(η−*x*/*v*) exp{−*J*4π*f*_0_*RG*(*x*, *y*, η)/*c*} *TRN*(τ−2*RG*(*x*, *y*, η)/*c*), which is the convolution kernel. In discrete format, Equation (3) can be written as:

(4) $$Y\left(\tau_{ir},\eta_{ia}\right)=\sum_{ja=1}^{N_{a}}\sum_{jr=1}^{N_{r}}A\left(ir,ia,jr,ja\right)X\left(x_{jr},y_{ja}\right)+N\left(\tau_{ir},\eta_{ia}\right)$$

where *Y*(τ*_ir_*, η*_ia_*) is the *ir*-th fast time sample at the *ia*-th slow time observation of the echo, *X*(*x_jr_*, *y_ja_*) is the backscattering coefficient at the *jr*-th position of the slant range and the *ja*-th position along the azimuth direction [9]. In matrix format, Equation (4) becomes:

(5) $$\bar{Y}=\bar{A}\bar{X}+\bar{N}$$

in which the convolution matrix can be expressed as:

$$\bar{\text{A}}=\left(\begin{array}{ccc} A_{\left(1,1\right)}\left(1,1\right) & \ldots & A_{\left(IX,IY\right)}\left(1,1\right)\\ A_{\left(1,1\right)}\left(1,2\right) & \ldots & A_{\left(IX,IY\right)}\left(1,2\right)\\ \vdots & \ddots & \vdots \\ A_{\left(1,1\right)}\left(1,\text{L}\right) & \ldots & A_{\left(IX,IY\right)}\left(1,\text{L}\right)\\ A_{\left(1,1\right)}\left(2,1\right) & \ldots & A_{\left(IX,IY\right)}\left(2,1\right)\\ \vdots & \ddots & \vdots \\ A_{\left(1,1\right)}\left(Q,L\right) & \ldots & A_{\left(IX,IY\right)}\left(Q,L\right) \end{array}\right)$$

with *A*_(_*_ix_*_,_*_iy_*_)_(*q*, *l*) = *w_a_*(η*_q_ − x_ix_*/*v*) exp{−*J*4π*f*_0_*RG*(*x_ix_*, *y_iy_*, η*_q_*)/*c*} *TRN*(τ*_l_* − 2*RG*(*x_ix_*, *y_iy_*, η*_q_*)/*c*), where τ*_l_*(*l* = 1,…, *L*) and η*_q_*(*q* = 1,…, *Q*) are the sampling time indicators at the range and azimuth directions, respectively; *ix* and *iy* index the grid positions (*i.e.*, coordinates along the azimuth and range directions) of the scene, respectively; and *IX* and *IY* indicate the maximum numbers of grid nodes along the azimuth and range directions, respectively

To facilitate the discussion of CS-radar using common notations in CS, we can form *n*(*n* = *IX* × *IY*) by 1 column vectors **Y***_n_*_×1_, **X***_n_*_×1_, and **N***_n_*_×1_ from their matrix formats by row-stacking (e.g., the first row of **X̄** is transposed and becomes the top *IX* elements of **X***_n_*_×1_, and subsequent rows are transposed and placed underneath). Thus, Equation (5) can be re-written:

(6) $${\text{Y}}_{n\times 1}={\text{A}}_{n\times n}{\text{X}}_{n\times 1}+{\text{N}}_{n\times 1}$$

where **A***_n×n_* is a block circulant matrix. Without causing any confusion, we may use **X**, **Y**, **N** and **A** for **Y***_n_*_×1_, **X***_n_*_×1_, **N***_n_*_×1_ and **A***_n_*_×_*_n_*, respectively, by omitting the subscripts shown in Equation (6).

Radar imaging refers to the process by which radar reflectivity **X** is reconstructed from echo data **Y**. This can be accomplished using the so-called range-Doppler algorithm [39]. For a CS-based radar imaging system, the number of samples collected in the receiver can be reduced (so, the lengths of column vectors **Y** and **N** and the number of rows of matrix **A** are denoted *m* (*m* < *n*) below), while a full-rank measurement matrix A is employed for a conventional radar imaging scenario. Thus, a set of CS-radar measurements can be written as:

(7) $${\text{Y}}_{m\times 1}={\text{A}}_{m\times n}{\text{X}}_{n\times 1}+{\text{N}}_{m\times 1}$$

where, again, the subscripts indicating the dimensions of the vectors and matrix concerned may be omitted, without causing any ambiguity, leading to a typical linear CS system: **Y** = **AX** + **N**.

As mentioned previously, there are typically three components in CS: sparse signals **X** (**X** ∈ ℝ*^n^*^×1^, e.g., a radar scene littered with only a few point-like objects (*i.e.*, targets) or dominated with a small number of targets with very strong reflectivity in contrast to a background of weak reflectivity), an information sampling mechanism (*i.e.*, an encoder) through a measurement matrix **A** (**A** ∈ ℝ*^m^*^×^*^n^*) to get under-sampled measurements **Y** = **AX** + **N** (**Y**, **N** ∈ ℝ*^m^*^×1^), which should be reasonably efficient in conveying information about **X**, and a signal reconstruction algorithm *χ̂* (*i.e.*, a decoder) that can detect sparsity patterns and/or estimate significant coefficients **X̂** from under-sampled data **Y** [4,6,7,40]. Consider the first two elements of CS briefly below. A sparse *n*-vector signal **X** = (*X*_1_, *X*_2_,…, *X_n_*)*^T^* (where superscript^T^ denotes transpose) means that it can be represented as **X** = **ΨΘ**, where **Ψ**(*n*×*n*) is the basis, and there are only *k* non-zero (or significantly different from zero) components (coefficients) in **Θ** (*k* ≪ *n*, with signal **X** called *k*-sparse) [2,3]. For an originally-sparse signal **X**, we may consider the basis as the identity matrix. CS sampling based on a linear system **Y** = **AX** + **N** says that measurement vector **Y** is a projection of signal **X** on the basis (*i.e.*, the columns of measurement matrix **A**) and contaminated with noise vector **N**. The compressibility of **X** and the trans-informational capacity of **A** can be analyzed in light of information theory, although radar scenes are typically noise-like and do not lend themselves to a straightforward compressive framework (so, some assumptions are necessarily made regarding the kind of radar scenes where a CS strategy is justified), as we explain next.

### 2.2. Informational Analysis of Compressive Radar Measurements

Below, we first describe suitable models for representing radar scenes and then their rate distortion functions. This is followed by a description of the method for estimating trans-information of a set of compressive samples about the underlying scene. Quantification of the rate distortion of a scene and trans-information of a set of measurements facilitates the determination of necessary undersampling ratios for scene reconstruction (*i.e.*, imaging), given certain scene sparsity, per-sample SNRs and tolerable distortion.

The underlying signal **X** can be considered as being discrete or continuous to accommodate the task of detection or estimation, respectively. We focus on the latter, bearing in mind that the former can be seen as a special case of the latter. As described by Oliver and Quegan [35], radar reflectivity is usually represented as complex-valued random variables having i.i.d. real and imaginary parts that are both modeled as Gaussian distribution of zero means and variance, indicative of radar image intensity, due to the large number of sub-pixel objects and their incoherently interfering reflectivities.

There are two characteristics of radar scenes (and their images) relevant to the discussion here: the noise-likeness and very high dynamic ranges. The former refers to speckle (which can be modeled as multiplicative exponential noise) and the white noise-like phase (which is uniformly distributed in [−π, π]) [35], while the latter is caused by the presence of a few bright objects in a scene [36]. Because of their noise-like properties and, hence, high entropy, complex-valued radar images are inherently difficult to compress efficiently (*i.e.*, they have limited compressibility in any dictionary). Despite these, the presence of corner reflectors, such as man-made structures, in a scene means that images containing such objects of strong reflectivity have very bright pixels localized on these objects, while the background of the image is much darker. This is to say that the pixels of strong-reflectivity objects in a radar image can be several orders of magnitude brighter than the background pixels [36]. Therefore, sparsity can be justified for radar scenes where there is only a small number of point-like strongly reflecting scatters or for radar surveillance applications, whereby the interest is in the detection of a few dominant objects (e.g., vehicles, ships or airplanes) [6,7]. In other words, we can assert that these objects are sparse in the geographic space due to their relatively small number and very strong reflectivities and that such radar scenes are sparse, at least, approximately, given the fact that our aim is often to detect and estimate these brighter objects against an otherwise darker background, that the radar raw data are contaminated with noise and that reconstructed sparse images need only to be accurate enough (in terms of the distortion threshold) for the purpose, even if they are approximately reconstructed.

Consider the image under study. We can denote the complex-valued image **X** ∈ **(IMAGE)***^n′^*^×1^; alternatively, it can be denoted **X** ∈ ℝ*^n^*^×1^ (*n* = 2*n′*) for consistency with the previous notations concerning the vector dimension without causing ambiguity if we use real representations for real and imaginary parts of **X** (which will be further discussed at the start of Section 3). The image **X** can be decomposed into two components: **X** = **X**_1_ + **X**_0_, where **X**_1_ represents the sparse bright objects and **X**_0_ the remaining background, as discussed by Rilling *et al.* [36]. This means that the image is the union of two disjoint sets: the sparse set and the non-spare set. The underlying scene **X** can thus be modeled by assuming a Gaussian mixture distribution: the n-dimensional vector **X** is a sequence of {*X_1_*,…,*X_n_*} drawn i.i.d. from a Gaussian mixture distribution:

(8) $$P_{x}∼\left(1-\kappa \right)N\left(m_{0},\sigma_{0}^{2}\right)+\kappa N\left(m_{1},\sigma_{1}^{2}\right)$$

where *κ* ≤ 1/2 indicates sparsity (*i.e.*, *κ* = *k*/*n*), *m*_1_ = *m*_0_ = 0, σ_1_ ≠ σ_0_ [33,41-43]. Gaussian mixture models were used for modeling natural images, where a problem domain is assumed to consist of homogeneous patches, each of which is assumed to be a Gaussian distribution with its own mean and variance [44]). Finite mixture models (for three classes of objects) were also employed for estimating the proportions of areas belonging to different classes in an SAR image without first segmenting it [45]. For a strictly sparse (or spike) signal, σ_0_ = 0, $V_{X}=\kappa \sigma_{1}^{2}$ (*V_X_* stands for the variance of a random variable (RV) *X* drawn from the vector **X**), which is reduced to the Bernoulli-Gaussian model discussed by Weidmann and Vetterli [46].

The entropy *H*(*X*) for an RV *X* is its minimum descriptive complexity and sets its ultimate limit to data compression if it were to be compressed and then decompressed without loss of information. For lossy data compression, as in CS-based radar imaging, a more useful quantity is the rate distortion function [22]. The rate distortion function *R*(*D*) of a source *X* determines the minimal number of bits per symbol, as measured by the rate *R*, that should be communicated over a channel, so that the source (input) can be reconstructed approximately (with the average distortion less than a given threshold *D*) at the receiver (output). The operational definition is equivalent to the information rate distortion function *R*^(^*^I^*^)^(*D*):

(9) $$R\left(D\right)=R^{\left(I\right)}\left(D\right)=\underset{p\left(\hat{x}|x\right):E\left(d\left(x,\hat{x}\right)\right)\leq D}{\min}I\left(X;\hat{X}\right)$$

where *X̂* represents the reconstruction of a random variable defined via the conditional probability mass (or density) function *p*(*x̂*|*x*), *I*(*X*; *X*) the mutual information between *X* and *X̂*, *d*(*x*, *x̂*) the distortion measure (a mean squared error (MSE) measure is used for a continuous signal X) and *E*(*d*(*x, x̂*)) the expected distortion obtained over the joint distribution of *p*(*x*, *x̂*) [22,46]. Reznic *et al.* [47] studied the rate distortion function for a mixture of two Gaussian sources as in Equation (8):

(10) $$R\left(D\right)=\{\begin{array}{ll} H\left(\kappa \right)+\frac{1-\kappa }{2}\log\left[\sigma_{0}^{2}/D\right]+\frac{\kappa }{2}\log\left[\sigma_{1}^{2}/D\right], & if\;D<\sigma_{0}^{2}\\ H\left(\kappa \right)+\frac{\kappa }{2}\log\left[\kappa \sigma_{1}^{2}/\left[D-\left(1-\kappa \right)\sigma_{0}^{2}\right]\right], & if\;\sigma_{0}^{2}<D\leq \left(1-\kappa \right)\sigma_{0}^{2}+\kappa \sigma_{1}^{2} \end{array}$$

where *H*(*κ*) is binary entropy. For a strict sparsity model, $\sigma_{0}^{2}\to 0$, we have:

(11) $$\begin{array}{cc} R\left(D\right)=H\left(\kappa \right)+\frac{\kappa }{2}\log\left[\kappa \sigma_{1}^{2}/D\right], & \text{if}\; \end{array}0<D\leq \kappa \sigma_{1}^{2}$$

as also shown in Aeron *et al.* [33].

Consider mutual information conveyed by radar measurements **Y** about the underlying scene **X** as in Equation (7). Here, we have a deterministic matrix **A** ∈ ℝ*^m^*^×^*^n^*, whose rows are denoted **A***_i_* (*i* = 1,…,*m*), and the noise vector **N** consists of a sequence of i.i.d. Gaussian RVs with variance *V_N_*. Instead of assuming each row of **A** restricted to having a unit *ℓ*_2_ norm, *i.e.*, ${\text{A}}_{i}^{\text{T}}{\text{A}}_{i}=1$, **A**_*i*_ does not necessarily have either unit or equal *ℓ*_2_ norm, but is only subject to having finite *ℓ*_2_ norm.

By definition, conditional mutual information between **X** and **Y**:

(12) I(X;Y|A)=h(Y|A)−h(N)

Furthermore, we can derive the inequality:

(13) $$h\left(\text{Y}|\text{A}\right)\leq h\left(\text{Y}\right)\leq h\left({\text{Y}}^{*}\right)$$

where Y* = AX* + **N** is a column vector of Gaussian RVs, X* is a vector of i.i.d. Gaussian RVs and has the same covariance as **X** and the inequality *h*(**Y**) ≤ *h*(Y*) originates from the fact that Gaussian RVs maximize entropies of distributions with the same variance. Further, we can put an upper bound on the joint entropy of Y*:

(14) $$h\left(\text{Y}*\right)\leq h\left(Y_{1}^{*}\right)+\sum_{i=1}^{m-1}h\left(Y_{i+1}^{*}|Y_{i}^{*}\right)\leq h\left(Y_{1}^{*}\right)+\sum_{i=1}^{m-1}h\left(Y_{i+1}^{*}-{\hat{Y}}_{i+1}^{*}\right)$$

where ${\hat{Y}}_{i+1}^{*}=b_{i}Y_{i}^{*}$ is the minimum mean squared error (MMSE) estimate for $Y_{i+1}^{*}$, with $b_{i}=\mathrm{cov}\left(Y_{i}^{*},Y_{i+1}^{*}\right)/\mathrm{var}\left(Y_{i}^{*}\right)$, where $\mathrm{cov}\left(Y_{i}^{*},Y_{i+1}^{*}\right)={\text{A}}_{i}^{T}{\text{A}}_{i+1}V_{X}$ and $\mathrm{var}\left(Y_{i}^{*}\right)={\text{A}}_{i}^{T}{\text{A}}_{i}V_{X}+V_{N}$. The variance for the error of ${\hat{Y}}_{i+1}^{*}$ (*i.e.*, $Y_{i+1}^{*}-{\hat{Y}}_{i+1}^{*}$, shown in the right-hand side of Equation (14)) is evaluated as:

(15) $$\begin{array}{ll} \mathrm{var}\left(Y_{i+1}^{*}-{\hat{Y}}_{i+1}^{*}\right) & =\mathrm{var}\left(Y_{i+1}^{*}\right)-2b_{i}\mathrm{cov}\left(Y_{i+1}^{*},Y_{i}^{*}\right)+b_{i}^{2}\mathrm{var}\left(Y_{i}^{*}\right)\\ & ={\text{A}}_{i+1}^{\text{T}}{\text{A}}_{i+1}V_{X}+V_{N}-2\frac{{\text{A}}_{i}^{\text{T}}{\text{A}}_{i+1}V_{X}}{{\text{A}}_{i}^{\text{T}}{\text{A}}_{i}V_{X}+V_{N}}\left({\text{A}}_{i}^{\text{T}}{\text{A}}_{i+1}V_{X}\right)+\frac{{\left({\text{A}}_{i}^{\text{T}}{\text{A}}_{i+1}V_{X}\right)}^{2}}{{\text{A}}_{i}^{\text{T}}{\text{A}}_{i}V_{X}+V_{N}}\\ & ={\text{A}}_{i+1}^{\text{T}}{\text{A}}_{i+1}V_{X}+V_{N}-\frac{{\left({\text{A}}_{i}^{\text{T}}{\text{A}}_{i+1}V_{X}\right)}^{2}}{{\text{A}}_{i}^{\text{T}}{\text{A}}_{i}V_{X}+V_{N}} \end{array}$$

from which we can quantify the upper bound of mutual information between **X** and **Y** as:

(16) $$\begin{array}{ll} I\left(\text{X};\text{Y}|\text{A}\right)=h\left(\text{Y}|\text{A}\right)-h\left(\text{N}\right) & \leq h\left({\text{Y}}^{*}\right)-h\left(\text{N}\right)=I{\left(\text{X};\text{Y}|\text{A}\right)}_{\text{ub}}\\ & =\frac{1}{2}\log\left({\text{A}}_{1}^{\text{T}}{\text{A}}_{1}\text{snr}+1\right)+\\ & \;\frac{1}{2}\overset{m-1}{\underset{i=1}{\text{∑}}}\log\left[{\text{A}}_{i+1}^{\text{T}}{\text{A}}_{i+1}\text{snr}+1-\frac{{\left({\text{A}}_{i}^{\text{T}}{\text{A}}_{i+1}\text{snr}\right)}^{2}}{{\text{A}}_{i}^{\text{T}}{\text{A}}_{i}\text{snr}+1}\right] \end{array}$$

where *I*(**X**; **Y**|**A**)_ub_ represents the upper bound for *I*(**X**; **Y**|**A**) and *snr* is the ratio of the variance of **X** over that of noise **N**. Note that we can compute the per-sample signal-to-noise ratio: $\text{SNR}=\frac{E\left[{\left\|\text{AX}\right\|}_{2}^{2}\right]}{E\left[{\left|\text{N}\right\|}_{2}^{2}\right]}$.

We may derive an approximate bound on the undersampling ratio *m*/*n* by requiring mutual information to be no less than *nR*(*D*):

(17) $$I{\left(\text{X};\text{Y}|\text{A}\right)}_{\text{ub}}\geq nR\left(D\right)$$

As *m* is generally implied in Equations (16) and (17), we can only use numerical methods to find critical values of *m* from this non-linear inequality, although an under-estimated sampling rate (in the case of zero correlation and constant *ℓ*_2_ norm for each row of **A**) is:

(18) $$m/n\geq R\left(D\right)/\log\left[1+\kappa \text{SNR}\right]=\left[H\left(\kappa \right)+\frac{\kappa }{2}\log\left(\kappa \sigma_{1}^{2}/D\right)\right]/\log\left[1+\kappa \text{SNR}\right]$$

where $0<D<\kappa \sigma_{1}^{2}$.

Mutual information *I*(**X**; **Y**|**A**) measures the amount of information conveyed by measurements **Y** about the signal **X** being estimated. Its upper bound quantified in Equation (16) sets an upper limit to the trans-information of **Y** about **X** and, hence, the amount of information retained in image **X̂** reconstructed from **Y** (*i.e.*, *I*(**X**; **X̂**|**A**). This follows the well-known data processing theorem in information theory, which states that *I*(**X**; **X̂**|**A**) ≤ *I*(**X**; **Y**|**A**) as **X** → **Y** → **X̂** forms a Markov chain [22]. Therefore, in addition to its importance for determining necessary sampling ratios, the amount of trans-information of compressive samples **Y** about **X** is also a valuable indicator (informational limit) for the resultant reconstructed image **X̂**, whether **X̂** is derived from convex optimization-based CS algorithms or not.

## 3. Results with Simulated Data

Radar images are complex-valued and contain information not only in amplitude, but also in phase [34,35,37,38]. To implement CS with radar imaging, we can use real representations for the complex-valued radar images [48], so that the CS techniques designed for real-valued signals can be employed, given that analysis and algorithms for complex signals are not well developed. Thus, complex-valued matrix **A** and vectors **Y**, **X** and **N** need to be decomposed into their real and imagery parts:

(19) $$\begin{array}{cccc} \tilde{\text{A}}=\left(\begin{array}{cc} ℜ\left(\text{A}\right) & -ℑ\left(\text{A}\right)\\ ℑ\left(\text{A}\right) & ℜ\left(\text{A}\right) \end{array}\right), & \tilde{\text{Y}}=\left(\begin{array}{c} ℜ\left(\text{Y}\right)\\ ℑ\left(Y\right) \end{array}\right), & \tilde{\text{X}}=\left(\begin{array}{c} ℜ\left(\text{X}\right)\\ ℑ\left(X\right) \end{array}\right), & \tilde{\text{N}}=\left(\begin{array}{c} ℜ\left(\text{N}\right)\\ ℑ\left(N\right) \end{array}\right) \end{array}$$

From these, we can formulate the CS-radar measurement model:

(20) $$\tilde{Y}=\tilde{A}\tilde{X}+\tilde{N}$$

CS-based radar imaging is formulated as:

(21) $$\begin{array}{ccc} \arg\underset{\tilde{X}}{\min}{\left\|\tilde{X}\right\|}_{0} & \text{s}.\text{t}. & {\left\|\tilde{Y}-\tilde{A}\tilde{X}\right\|}_{2}\leq \varepsilon \end{array}$$

where *ε* > 0 For solving Equation (21) *ℓ_q_*-minimization algorithms, such as orthogonal matching pursuit (OMP) [49,50] and compressive sampling matching pursuit (CoSaMP) [51], can be implemented [9], although a detailed discussion of signal reconstruction is beyond the scope of the paper. Below, we describe simulation-based experimental results and provide some discussion.

The simulation-based experiment over a single transect of a ground range of 1000 m proceeded as follows:

(1) specify radar parameters as indicated in Equation (3) (slant range of scene center 4472 m, altitude 4000 m, transmitted pulse duration 2 (μs, range FM rate 50 MHz/(μs, signal bandwidth 100 MHz, range sampling rate 100 MHz, range sampling swath width 1000 m (resulting in 498 range cells), effective radar velocity 100 m/s, radar center frequency 3.0 GHz, azimuth FM rate 44.7214 Hz/s, azimuth sampling rate 46 Hz) to generate a convolution kernel matrix A;
(2) set up a series of sparsity (equally spaced in the interval 0.01–0.20, step = 0.0025), undersampling ratios (equally spaced in the interval 0.05 ∼ 1, step = 0.0128, SNR (equally spaced in the interval −5 dB∼20 dB, step = 0.3378 dB) and distortion levels *D* (three values, 0.01, 0.02, 0.04, to represent relatively high, moderate and low accuracy levels, respectively, although many more levels of distortion can be specified in principle);
(3) simulate scene **X** (*i.e.*, radar reflectivity) and then noise **N** to get simulated radar echo data **Y** (**Y** = **AX** + **N**) using the convolution kernel matrix **A** simulated in Step 1; simulation of **X** is performed with specific sparsity in the mixture model (Equation (8)) and assuming unit variance of sparse objects in an assumed strictly sparse scene, while **N** is simulated with a series of variance values inversely proportionate to the series of SNR values ( $\text{SNR}=\frac{E\left[{\left\|\text{AX}\right\|}_{2}^{2}\right]}{E\left[\left[{\left\|\text{N}\right\|}_{2}^{2}\right]\right]}$) specified in Step 2;
(4) perform informational analysis of simulated radar sparse scenes and radar measurements, such as *R*(*D*) (Equations (10) or (11), depending on whether an approximately or strictly sparse scene is simulated; here, we use the latter, as the strictly sparse scene **X** is simulated) and I(**X**; **Y**|**A**) (actually *I*(**X**; **Y**|**A**)_ub_ in Equation (16)) and determine minimal undersampling ratios for signal reconstruction given certain values of sparsity, SNR and distortion *D* (according to the condition in Equation (17));
(5) run CS reconstruction algorithms, such as CoSaMP [51], to recover **X** from **Y** (*i.e.*, to derive **X̂** from undersampled data **Y**);
(6) generate phase transition diagrams, where the image quality of **X̂** is assessed in terms of the probability that relative errors between **X̂** and **X** are within the threshold of 1/3.

Step 3 is explained in more detail below. For each set of sparsity, undersampling ratio and SNR, we generated a set of realized scene **X**, noise **N** and echo data **Y** (as **Y** = **AX** + **N**, given measurement matrix A specified above). Specifically, the complex-valued scene **X** was generated using the Gaussian mixture-based sparsity model (with the real and imaginary parts simulated independently); noiseless linear measurements **Y**_0_ were generated by pre-multiplying the simulated scene **X** with randomly-drawn rows from the measurement matrix **A**; the measurements **Y_0_** were corrupted with additive Gaussian noise N, whose power is restricted to the given SNR level, to simulate noisy and undersampled data **Y**.

**Figure 1:**
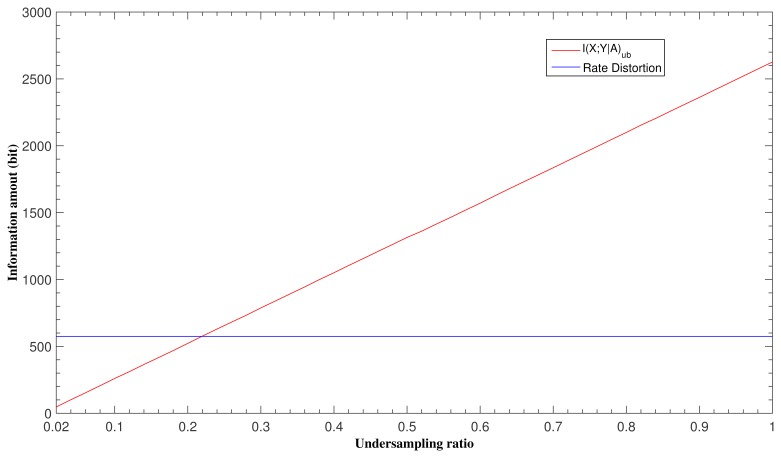
Rate distortion and upper bound for mutual information under different undersampling ratio, with sparsity, SNR and distortion fixed as 0.2, 10 dB, 0.01, respectively.

Mutual information *I*(**X**; **Y**|**A**) measures the amount of information conveyed by measurements **Y** about the scene **X** being reconstructed (*i.e.*, the trans-information of **Y** about **X**), as discussed previously. Figure 1 shows the behaviors of the rate distortion function and mutual information *I*(**X**;)_ub_ in relation to undersampling ratios. For example, for a simulated sparse signal **X** (sparsity = 0.2), SNR = 10 dB and distortion *D*= 0.01, the rate distortion (*n* * *R*(*D*)) of information source **X** can be calculated using Equation (11), while *I*(**X**; )_ub_ can be determined by Equation (16) given undersampling ratios ranging from 2% to 100% (equally spaced with a step of 2%). The upper bound for mutual information *I*(**X**; )_ub_ increases approximately linearly with undersampling ratios and exceeds the rate distortion threshold when the undersampling ratios get greater than 23%, as shown in Figure 1; this means that there would be information loss if **Y** were undersampled below 23% (i.e., reconstruction errors for scene **X** would be greater than what distortion level *D* would imply), while there is steady information gain in **Y** about **X** with increasing sampling ratios. This explains an important assertion in CS that one only needs to get enough (in terms of information content pertaining to **X**) samples for reconstruction of a scene **X** (containing a certain amount of information H(**X**), but requiring only some minimum information rate *R*(*D*) for its lossy compression). Below the critical threshold dictated by *R*(*D*), the sophistication of the CS signal reconstruction algorithms is no substitution for adequate information-laden samples.

**Figure 2:**
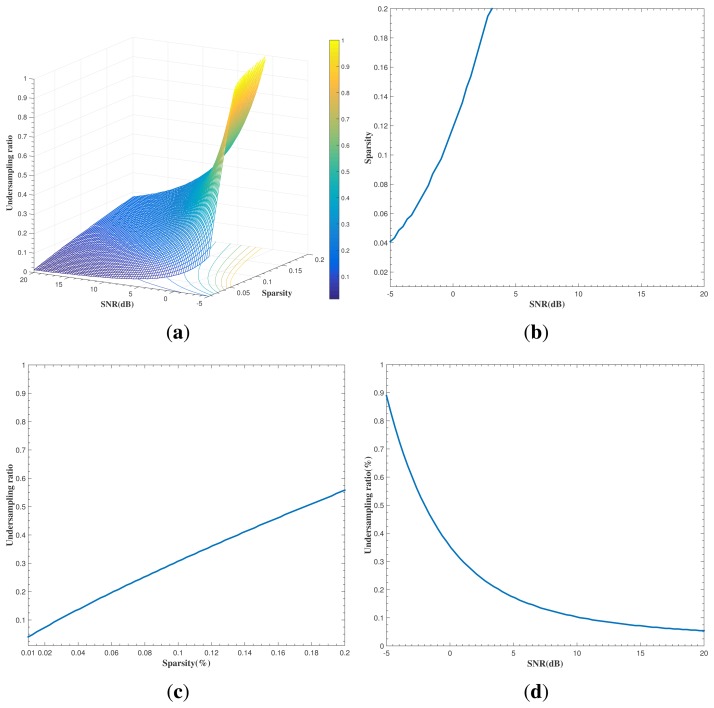
Information-theoretically-derived relationships among sparsity, SNR and undersampling ratios (distortion level = 0.01): (**a**) necessary undersampling ratios in relation to scene sparsity and SNR; (**b**) sparsity-SNR relationship with undersampling ratio = 75%; (**c**) sparsity-undersampling ratio relationship with SNR = 5 dB; and (**d**) undersampling ratio-SNR relationship with sparsity = 0.05.

The minimal undersampling ratios for a given signal sparsity and SNR can be determined through evaluating the information-theoretic inequality in Equation (17). This can be done by numerically solving an equation between the mutual information *I*(**X**; **Y**|**A**)_ub_ and rate distortion implied in Equation (17). Figure 2a shows the surface of minimal undersampling ratios in relation to scene sparsity and per-sample SNR, given a distortion level of 0.01. Obviously, different surfaces depicting necessary undersampling ratios given scene sparsity and per-sample SNR can be generated depending on the specific MSE distortion level required.

For two-dimensional visualization of these three quantities, we can set a specific value for one quantity and carve out a curve depicting the relationship between the other two. Figure 2b–d show the sparsity-SNR curve with undersampling ratio = 75%, the sparsity-undersampling ratio curve with SNR = 5 dB and the undersampling-SNR curve with sparsity = 5%, respectively.

**Figure 3:**
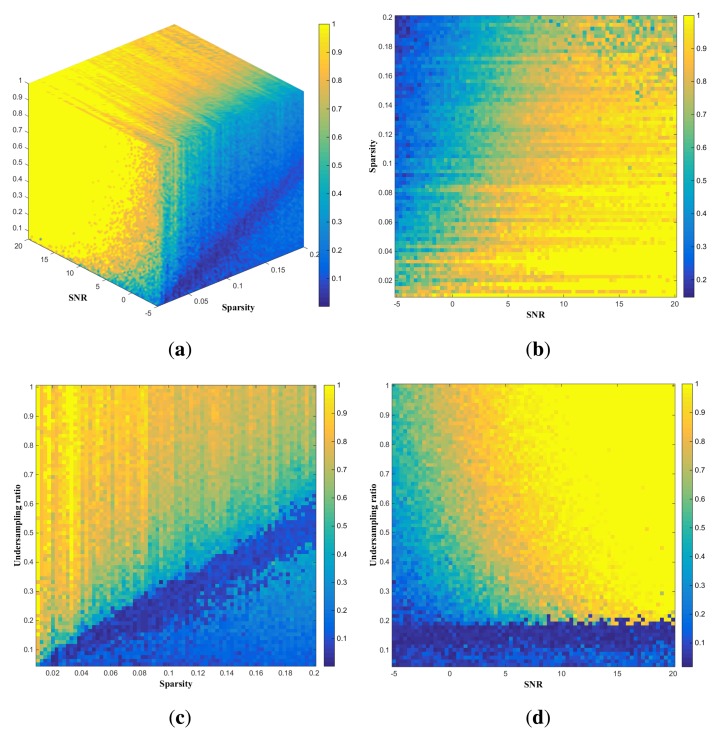
Computationally-derived relationships among sparsity, SNR and undersampling ratios (distortion level = 0.01): (**a**) necessary undersampling ratios in relation to scene sparsity and SNR; (**b**) sparsity-SNR relationship with undersampling ratio = 75%; (c) sparsity-undersampling ratio relationship with SNR = 5 dB; and (**d**) undersampling ratio-SNR relationship with sparsity = 0.05.

To complement and validate the theoretically-derived results shown in Figure 2, we carried out computational experiments by simulating and reconstructing a large number of scenes and summarizing the probability of successful scene reconstruction on the criterion that the relative error between **X̂** and **X** is within 1/3. Each realization of **X** corresponds to a particular sparsity, while each corresponding undersampled **Y** is the result of a particular combination of **X**, the undersampling ratio and SNR, as shown previously. To identify the necessary number of samples of **Y** for reconstructing **X**, we used the sparse signal reconstruction algorithm CoSaMP mentioned previously to recover **X** from **Y** (*i.e.*, to derive **X̂** from undersampled **Y**) and summarized the results in terms of the probability of successful reconstruction. This resulted in the so-called phase diagram shown in Figure 3a, where different colors in the undersampling ratio-sparsity-SNR space indicate varying levels of probability in successful scene reconstruction. Figures 2b-d and 3b-d show the sparsity-SNR relation with the undersampling ratio = 75%, the sparsity-undersampling ratio relation with SNR = 5 dB and the undersampling-SNR relation with sparsity = 5%, respectively.

In the remainder of this section, we first compare the theoretically-derived results shown in Figure 2 with the computational results in Figure 3, highlighting their differences, while acknowledging their complementarity. Then, we compare the results obtained in this paper with some relevant ones in the literature, in particular those concerning phase diagrams [9,25] and CS-radar performances [5,6], respectively, to emphasize the advantages of the proposed methods relative to those in the literature.

The comparison between Figures 2 and 3 indicates that information-theoretic necessity undersampling ratios (Figure 2a) seem under-estimated relative to those derived computationally, as shown in Figure 3a. This is first because of the fact that information-theoretic results should be interpreted on probabilistic

ground: *I*(**X**; **Y**|**A**) and *R*(*D*) are expectations, and the condition that information conveyed by **Y** should exceed *n* * *R*(*D*) is to ensure that the reconstruction error of **X** by **Y** will be within the distortion level *D* on average. The other reason is that the amount of mutual information estimated by Equation (16) is actually an upper bound for the true value of *I*(**X**; **Y**|**A**), implying that the necessary undersampling ratios derived from using the quantity in Equation (17) will be an under-estimate of the actual ones.

We discuss the results obtained above further with respect to those in the existing literature, as mentioned previously. Donoho and Tanner [25] conducted extensive computational experiments finding sparse solutions to a large variety of system **Y** (**Y** = **AX**, with **X** being an *n*-vector having *k* nonzeros. Their computational results were depicted via fractions of successful reconstruction in an undersampling-sparsity (*m*/*n- k*/*m*) domain called phase space, resulting in a so-called phase diagram (two-dimensional). Zhaing *et al.* [9] undertook computational experiments to generate three-dimensional (*m*/*n-k*/*n-SNR*) phase diagrams in the context of radar imaging. The theoretically-derived phase diagram shown in Figures 2a (where the distortion level is implicitly incorporated as opposed to the noiseless setting in Donoho and Tanner [25]) and the computationally-derived version in Figure 3a can be generated and interpreted in combination, so that they are more informative about sampling-sparsity-SNR-distortion trade-offs than earlier results in Donoho and Tanner [25] and Zhang *et al.* [9].

After a discussion about the relative superiority of the proposed methods to generate phase diagrams to visualize the sampling-sparsity-SNR-distortion interdependencies in comparison with some of the relevant work, we elaborate on the significance of the proposed methods by relating them to some of the early research efforts on CS-radar and compressive radar imaging, in particular [5,6], after reviewing their relevant results.

In [5], the CS method was tested on one-dimensional simulated signals and with real SAR raw data that were used to form two-dimensional images. In the experiment with simulated data, 10% to 70% of the samples were randomly taken; the results shown in [5] were obtained by keeping 50% of the samples received. The convex linear problem involved in CS was solved with a regularized OMP algorithm ([50]). For both CS and MF, a scene with ten point targets was simulated, and tests were carried out with no noise, as well as with SNRs ranging from −10 to 20 dB (though results were presented for only three cases in [5]: 0, 10 and 20 dB). By comparison with the results obtained with the conventional matching filter method, it was shown that an image can be reconstructed, without loss of resolution, after dropping a large percentage of the received echo data. The results with real data in both an ocean scene and a more complex scene consisting of a mixture of sea, rural and urban surfaces showed promising performances of CS-based radar imaging when only 50% of the radar echo data was used. It was anticipated that CS techniques would allow the implementation of wide-swath modes without reducing the azimuth resolution [5].

Ender [6] described three possible applications of CS techniques: pulse compression, inverse SAR imaging and air space surveillance with array antennas. We focus on the results in [6] concerning pulse compression here. Both simulated data and real data acquired by an experimental radar system of Fraunhofer FHRwere employed in the experiments, although we review only the results with simulated data here. The simulated example was one-dimensional. With a sparsity of 15 dominant reflectors over 500 gridded points along the range, *m* = 100 sensing waveforms were drawn by random out of a total of *n* = 500 frequencies for illumination of the targets. For CS-based signal reconstruction, Ender [6] applied the simplex algorithm by transforming the original minimization problem (which could otherwise be solved by noisy basis pursuit algorithms to handle the case of noisy measurements) to the standard of the simplex algorithm. Again, to test the CS technique's robustness against noise, simulated noise data were added to simulated echo data with four SNR values (20, 30, 40 and 50 dB). As expected, the CS algorithm works perfectly with extremely high SNR, say 50 dB; its performance degenerates with an SNR of 20 dB or lower. The preliminary investigations on CS-radar promote further analysis and developments of suitable architectures and processors for enhancing radar performances.

In comparison, it can be seen that the CS-radar experiments reported in [5,6] are complementary to the information-theoretic analyses and assertion pursued by this paper. The complementarity is interpreted on the grounds that relevant results in [5,6] would be specific cases (*i.e.*, points) in the phase diagrams generated by the methods proposed in this paper if the scene characteristics, measurement/sensing matrices, undersampling ratios, noise and distortion were assumed to be the same as the corresponding cases in [5,6] and that what could be depicted by the phase diagrams generated in this paper are supposed to be able to exhaust all plausible scene-sensing configuration space in the context of compressive radar imaging (implicit of a particular distortion threshold *D* and conditional on sensing matrix **A**, as discussed further below). The significance of information-theoretic perspectives on CS-radar is also highlighted in [6], where the loss of information in CS induced by sparsity was measured through the use of the Fisher information matrix (and the derived Cramér–Rao bounds) for parameters, say range and direction, and the amplitudes and phases of distinct scatters. The complementarity of the work in [5,6] and here is also reflected in algorithmic aspects (although the algorithms are not the focus of the research in all three), as different algorithms adopted for scene reconstruction reveal not only differences in terms of the objective functions specified and the computational efficiency achieved, but also their limits to enhancing the quality of reconstructed images if necessary information redundancy (in terms of extra sampling required in addition to that dictated by rate distortion functions) is not allowed. This will be further discussed in the concluding section. Furthermore, experiments based on real data in [5,6] provide impetus for further developments in information-theoretic research on CS-radar, as promoted in this paper. Practical considerations will include treatment of radar clutters, comparisons with conventional SAR imaging and the computational efficiency required of operational CS-radar systems ([5,6]). However, the performances of compressive radar imaging experiments (simulated) in [5,6] and this paper are not comparable in rigorous terms due to a lack of commonality in terms of scene nature and geometry, the CS algorithms implemented and the waveforms (*i.e.*, sensing matrix A) adopted, as elaborated below.

As hinted above, both analytical and computational results obtained with simulated data are conditional on the particular measurement matrix **A** set forth, as is also the case with Zhang *et al.* [9]. In other words, our results are not invariant to the specificity of measurement matrices (and hence, the radar transmitted waveforms and other relevant parameters) employed in a CS system. This raises, for instance, the issue of how waveforms should be designed to maximize mutual information between Y and X, as discussed by Bell [19]. Clearly, the existing literature on related topics should be integrated for maximum benefits.

Classic information theory introduced by Shannon provides the mathematics for the design of the transmitter and receiver in a communication system to efficiently and reliably transmit the information from the source to the destination given the characteristics of the source and the channel. There was research carried out on information-theoretic analysis of radar systems before the advent of CS and CS-radar [17,19,52]. The results obtained in this paper reinforce the view that information-theoretic perspectives are constructive for system designs for traditional and CS-radar systems alike. Certainly, the informational quantities described in this paper in the context of CS-radar should be made to augment the existing predominantly statistical metrics for the performance evaluation of radar systems.

## 4. Conclusions

This paper provided the informational description, analysis and interpretation of information flows from the source (an often approximately sparse radar scene where the objects to detect and estimate are in small numbers, but possess distinctly strong reflectivity), through the measurements, to the destination (radar imaging). For this, the paper has clarified sparsity models and rate distortion functions that are applicable for CS-based radar imaging. As one of its key thrusts of innovation, the paper proposed a more realistic and accurate method for quantification of trans-information between compressive noisy measurements and the underlying sparse scene than previous studies. This was accomplished through derivation of the joint entropy of the undersampled data and mutual information conveyed by the undersampled data about the underlying scene in general formulas.

Past research has been directed either toward theoretical derivations of information quantities, such as measures of sparsity, rate distortion behaviors of sparse sources and sampling rates sufficient and/or necessary for sparsity recovery and estimation, or towards simulation-based studies of phase diagrams showing sampling rate-sparsity-SNR conditions for signal reconstruction. Very few studies have been done to not only validate theoretical results with experimental results, but also to provide information-theoretically-derived phase diagrams for guiding empirical implementations, especially in the context of radar imaging. This paper represents a rare piece of first work along this line by combining information-theoretic deduction of a few important informational quantities with simulation-based validation. The simulation results were found to be in close agreement with the theoretical results and will provide valuable information-theoretic insights for CS-radar system design and performance evaluation.

The results derived for continuous amplitude estimation can be extended to two scenarios: one concerning discrete support recovery, which requires a lesser amount of sampling, the other being conditions for exact (as opposed to approximate) reconstruction, although this is not elaborated in this paper. Further research needs to be carried out also to model and analyze the impacts of radar clutter on information flows and sampling rates required for scene reconstruction, because clutter interferences are common in radar remote sensing and should be properly handled in CS-radar for accuracy in target detection and estimation. Moreover, the specific algorithms for scene reconstruction are not considered in theoretical derivation, although computational results were dependent on them. Thus, we need also to quantify the extra information redundancy (more samples) required for algorithmic complexity and computing expenses, because information loss incurred in undersampling may not be compensated for by CS signal reconstruction algorithms, no matter how sophisticated or complicated they are. Lastly, experiments with real radar raw data are important for bridging the gap between CS theorems and their real-world practicality in radar imaging and for comparative testing and validation concerning conventional *vs.* CS-radar imaging strategies. For this, information theory plays an essential role in clarifying the (dis)advantages of both conventional and new strategies in terms of image quality and, more importantly, determining the limits of accuracy (e.g., MSE distortion) and resolution (both radiometric and spatial) through informational analysis.
